# Insecticidal and antifeedant activities of clerodane diterpenoids isolated from the Indian bhant tree, *Clerodendron infortunatum* , against the cotton bollworm, *Helicoverpa armigera*

**DOI:** 10.1093/jis/14.1.29

**Published:** 2014-01-01

**Authors:** Gholamreza Abbaszadeh, Chitra Srivastava, Suresh Walia

**Affiliations:** 1 Horticultural Research Institute, Shahid Bahonar University of Kerman, Iran; 2 Division of Entomology, Indian Agricultural Research Institute, New Delhi 110012, India; 3 Division of Agricultural Chemicals, Indian Agricultural Research Institute, New Delhi 110012, India

**Keywords:** bioassay, botanical biopesticides, phytochemicals, secondary metabolites

## Abstract

The Indian bhant tree, *Clerodendron infortunatum* L. (Lamialus: Lamiaceae), is a well-known medicinal plant, but little information about its bioefficacy against agricultural pests exists. This scarcity was addressed in the present study, in which dried leaves of *C. infortunatum* were subjected to extraction with hexane and methanol and then partitioned using different solvents of varying polarity. In a preliminary bioassay, the antifeedant effects of the crude extracts and fractions were tested on a highly polyphagous pest, the cotton bollworm, *Helicoverpa armigera* Hübner (Lepidoptera: Noctuidae), using the no-choice test method with cabbage leaf discs. The methanol fraction resulted in maximum antifeedant activity. This fraction was further subjected to crystallization and column chromatography in order to isolate the compounds responsible for the activity. Three pure compounds were isolated and identified as clerodin (CL), 15-methoxy-14, 15-dihydroclerodin (MD), and 15-hydroxy-14, 15-dihyroclerodin (HD). The antifeedant activity of these compounds was studied using a choice as well as a no-choice test method with 24 and 48 hr observation periods. Insecticidal activity was measured using the topical application method at 0.5, 1, 1.5, 2, 2.5, and 3% concentrations, and data were recorded 24, 48, and 72 hr after treatment. In the no-choice test conditions, compounds CL and MD showed significantly higher antifeedant activity compared to the key ingredient in many commercial pesticides, azadirachtin, at its highest concentration. Compound HD also showed very good antifeedant activity, which did not differ significantly from that of azadirachtin. In the choice test conditions, all three compounds and azadirachtin showed 100% antifeedant activity at the highest concentration. Antifeedant Index (AI50) values of CL, MD, and HD were 6, 6, and 8 ppm in choice tests, and increased to 8, 9, and 11 ppm in the no-choice tests, respectively. Insecticidal activity of the isolated compounds was not significant compared to the control condition, even at the highest con-concentrations of the compounds. These results suggest that extracts of *C. infortunatum* have very good antifeedant effects against *H. armigera* due to the presence of specific compounds. These compounds could be utilized in the development of new biopesticides.

## Introduction

Indiscriminate use of synthetic insecticides has led to problems such as the resurgence of primary pests, secondary pest outbreaks, resistance development, insecticide residue, health hazards, environmental contamination, and increased costs of insect control ( Roy and Mukhopadhyay 2010 ). One solution for these problems is utilization of plants’ bioactive molecules. Plants are the most efficient pro-ducers of phytochemicals in the environment, including secondary metabolites that are used by the plant in defense against phytophagous insects ( Ahmad 2007 ). These secondary metabolites include tannins, alkaloids, polyphenols, terpenoids, polyacetylenes, flavonoids, unusual amino acids, sugars, phenylpropanoids, quinines, essential oils, etc., that have a wide range of anti-insect properties, including insecticidal, repellent, antifeedant, and insect growth inhibitory activities ( Ahmad 2007 ; Dhaliwal and Koul 2011 ). These phytochemicals and plant extracts have been investigated intensively for the past 30 years in an effort to develop alternatives to conventional insecticides ( Isman 2006 ). The goal of research on plant secondary metabolites is to find new environment-friendly, biodegradable, and biologically-active natural products with low mammalian toxicity to avoid the deleterious effects of synthetic chemicals on the environment and nontarget animals ( Kubo 1997 ).

Terpenoids are a large class of natural products that includes various types, such as monoterpenes, sesquiterpenes, triterpenoids, and diterpenes. Considerable attention has been paid to the insect antifeedant activity of some natural clerodane diterpenoids isolated from several plant families ( Wagner et al. 1983 ). The Indian bhant tree, *Clerodendron infortunatum* L. (Lamiales: Lamiaceae), is an important medicinal plant. It is a terrestrial shrub with a disagreeable odor, and is common throughout the plains of India. Various parts of the plant have been used by native tribes to medicate colic, scorpion strings, snake bites, tumors, and certain skin diseases ( Ahmed et al. 2007 ). The present study was carried out to investigate the insecticidal and antifeedant activities of *C. infortunatum* against the cotton bollworm, *Helicoverpa armigera* Hübner (Lepidoptera: Noctuidae), a highly polyphagous pest, and to isolate the compounds responsible for such activities.

## Materials and Methods

### Plant material

Fresh leaves of *C. infortunatum* were collected from Behraich District of Uttar Pradesh, India, during October 2010 and identified by the Botanical Survey of India, Kolkata. The reference plant with voucher no. C.i.51/53 was kept in the Indian Agricultural Research Institute toxicology lab.

### Extraction of crude extracts

The leaves were dried in the shade at room temperature and then ground in an electric grinder. 2.5 kg of leaf powder was soaked in 2.5 L of n-hexane in a conical flask for 72 hr and shaken occasionally. The content of the conical flask was filtered using Whatman No.1 filter paper (Sigma-Aldrich, www.sigmaaldrich.com ), and the filtrate was concentrated in a vacuum using a rotary vacuum evaporator (Heidolph, www.cuisinetechnology.com ) at 40–42°C. The concentrated crude hexane extract was partitioned with hexane (to separate non-polar compounds) and methanol (to separate polar compounds), and concentrated into hexane and methanol fractions. Although methanol and hexane are not miscible, there is always the possibility that the polar compounds will be distributed, but not dissolved, in hexane; therefore, the methanol partition would separate polar compounds from hexane. After filtration, the leaf powder residue was further extracted with methanol and concentrated into crude methanol extract in a vacuum at 44– 45°C. This extract was further partitioned with hexane, ethyl acetate, and butanol, and concentrated into respective fractions.

### Isolation of active compounds

Based on the preliminary bioassay, the methanol fraction of hexane extract was dissolved in 50 mL of methanol and kept in a refrigerator for crystallization. Crystals were separated using a microfiltration assembly, weighed, and kept in the refrigerator for further use. The remaining residue of methanol fraction (10 g) was then subjected to silica gel (60–120 mesh) chromatography and eluted with hexane containing increasing amounts of ethyl acetate. Fractions were monitored on 0.25 mm pre-coated silica gel thin layer chromatography (TLC) plates 60 F254 (Merck Millipore, www.merckmillipore.com ), and spots were viewed under UV light. The fractions were then sprayed with a 20% sulfuric acid solution. The following ratios of mobile phase were used for fractionation:

**Tab:**
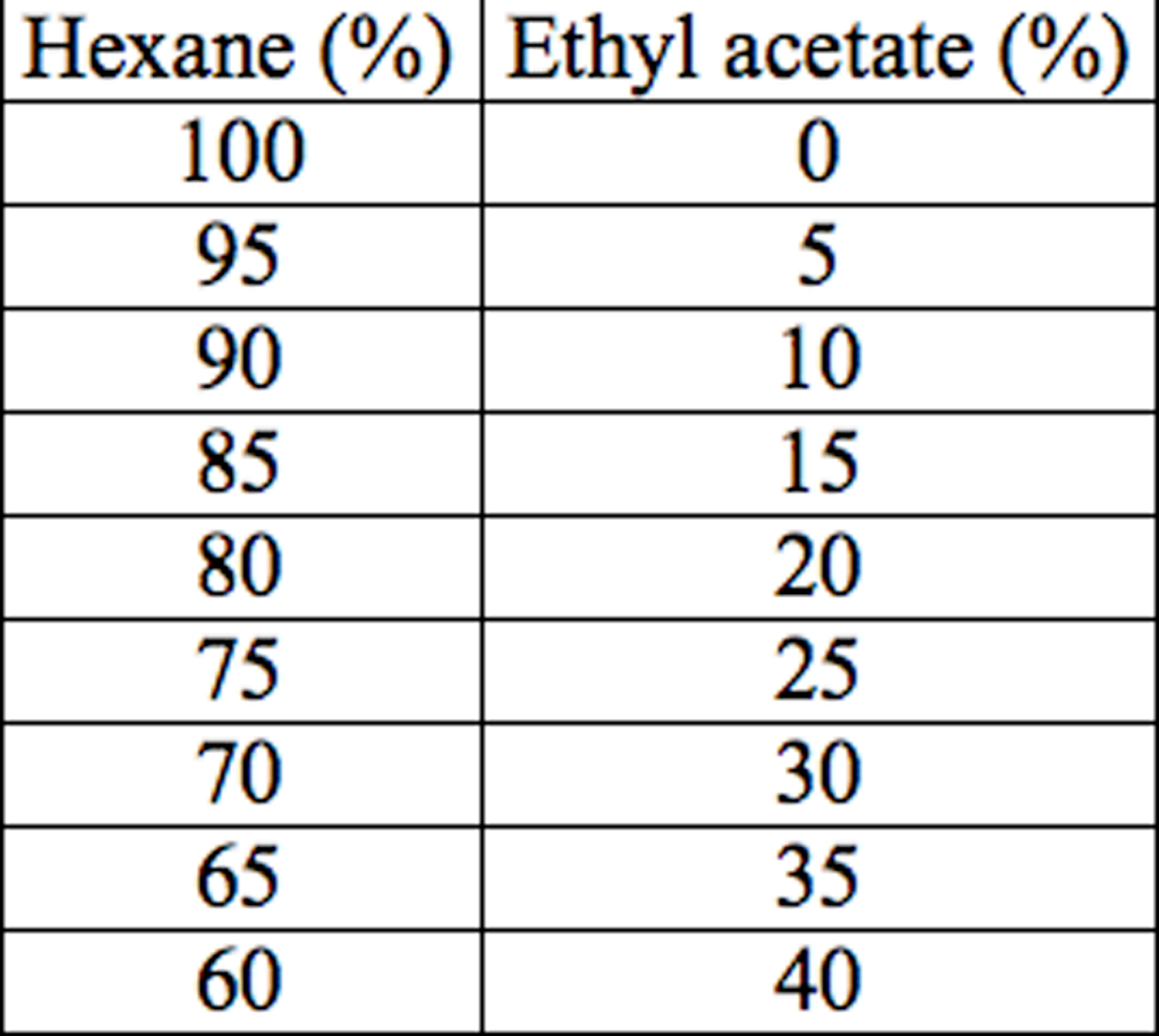


Nine fractions were collected and concentrated. Fractions six (75:25), eight (65:35), and nine (60:40) were revealed by TLC to each have a single spot, which were found to be pure compounds. The structure of these compounds was elucidated by spectroscopic methods, including data from nuclear magnetic resonance spectrometry (NMR), specifically ^1^ H-NMR and ^13^ C-NMR, and mass spectrometry (MS).

MS was carried out using an MS spectrometer (Thermo Fisher Scientific, www.thermofisher.com ). Detection of mass was done by electron-spray ionization source with a Finnigan LCQ tune plus program fitted with MAX-detector (Thermo Fisher Scientific). Xcalibur Software (Thermo Fisher Scientific) was used for identification, quantification, and fragmentation of required masses. The following MS parameters were optimized in direct infusion mode: spray voltage, 3.5–4.5 kV; sheath gas flow rate, 15–22 mL/min ^-1^ ; auxiliary gas flow rate, 0–2 mL/min ^-1^ ; spray current, 0.5; capillary temperature, 255–260°C; capillary voltage, 20–35 V; tube lens offset, 20–25. NMR spectrum of the compound was recorded on a 400 MHz spectrometer (Bruker, http://www.bruker.com ) using CDCl3 as solvent and tetra methyl silane as an internal standard. Chemical shifts were recorded in δ(ppm) values relative to tetra methyl silane. Analytical reverse phase high-performance liquid chromatography (HPLC) was performed with a 600 series pump and controller system equipped with a 996 photo diode array detector, a Rheodyne injector, and an RP-18 lichrospher column (3.9 mm id x 150 mm) (Waters, www.waters.com ). The system was run in isocratic mode at a flow rate of 0.75 mL/min. HPLC-grade acetonitrile and water (70:30) were used as mobile phase. A 20 µL volume of sample was injected each time via the Rheodyne injector. The samples were filtered through a 0.25 µm Millipore filter before injection. The calibration and quantification was carried out using Empower pro software (Waters).

### Test insects

Larvae of *H. armigera* were collected from chickpea fields in the Indian Agricultural Research Institute, New Delhi, India. The larvae were fed an artificial diet comprised of 720 mL double distilled water, 110 g chickpea powder, 20 g yeast powder, 10 g casein, 2 g methyl p-hydroxy benzoate, 0.5 g sorbic acid, 1 mL formaldehyde (40 %), 2.6 g ascorbic acid, 0.115 g cholesterol, 0.1 g streptomycin sulphate, 0.6 g vitamin E, 1 g multivitamin (Multivite ^R^ ), and 12 g agar. The larvae were fed in the laboratory at 26 ± 1°C, 13 ± 1 hr photophase, 11 ± 1 hr scotophase, and 75 ± 5% relative humidity. After emergence, adults were released into the oviposition chambers for egg laying and provided with 20% honey solution. Fresh, tender tomato leaves were kept inside the chambers to stimulate oviposition. The eggs were kept in hatching chambers at 75 ± 5% relative humidity. Newly-hatched larvae (neonates) were maintained on the artificial diet. Third instar larvae (weighing 30– 40 mg) were used for bioassay.

### Antifeedant activity

The antifeedant activities of different extracts and fractions were observed as a preliminary bioassay through a no-choice test method, and data were recorded after 24 hr. Based on this data, further purification was performed to isolate the pure compounds responsible for the activity. The antifeedant activity of the isolated pure compounds was studied using the leaf disc no-choice ( Singh and Pant 1980 ) and choice test methods ( Isman et al. 1990 ), and observations were recorded 24 and 48 hr after treatment. Fresh, tender leaf discs (4 cm diameter) of cabbage were used as food. Concentrations of the isolated pure compounds ranged from 10 to 5,000 ppm in the no-choice condition and 7–5,000 ppm in the choice condition. To make various concentrations, a 1% solution of plant material was dissolved into 100 mg of each compound in 10 mL of acetone. Other concentrations were also prepared by serial dilution and converted to ppm. The leaf discs treated with acetone were used as a negative control, and those treated with azadirachtin (20% purity) were used as a positive control. Wet filter paper was placed in each Petri dish (1.5 ×9 cm) to avoid early drying of the leaf disc. A single 3 ^rd^ instar larva of *H. armigera* was placed in each Petri dish. Five replicates, each comprised of five larvae, were maintained for each treatment condition. The bioassay was conducted in the same way in the choice and the no-choice test methods, except in the choice method there was one untreated leaf disc of the same size as the treated leaf disc that was kept in each Petri dish. The experiment was conducted under laboratory conditions of 26 ± 1°C, 14:10 L:D photoperiod, and 75 ± 5% relative humidity. Progressive consumption of leaf area by the larvae was recorded after 24 hr in the control and treated leaf discs using a LI-COR-3100 leaf area meter (LI-COR Biosciences, www.licor.com ).

The percentage of antifeedant activity in the no-choice test condition was calculated using the Singh and Pant (1980) formula:

$\text{Antifeedant activity} (\%) = (\% \text{protection in treatment} - \% \text{protection in control}) \times 100 / (100 - \% \text{protection in control}).$

The area of the leaf discs was measured by the leaf area meter before treatment as well as 24 and 48 hr after treatment; by having the leaf area before and after treatment, percent protection was able to be calculated and inserted into the formula. The percentage of antifeedant activity in the choice test condition was calculated using the Isman et al. (1990) formula:

$\text{Antifeedant activity} (\%) = (\text{C} - \text{T}) \times 100 / (\text{C} + \text{T})$

in which C = area consumed in control and T = area consumed in treatment.

### Insecticidal activity

The insecticidal activity of different extracts and azadirachtin against 3 ^rd^ instar larvae (30– 40 mg) was assessed via topical application of compounds to the *H. armigera* larvae. Stock solutions of the test compounds were prepared in acetone; further dilutions were done in emulsified water by maintaining the Triton ^TM^ X-100 emulsifier level at 0.5% in order to yield various concentrations (0.5–3%). 2 µL of different concentrations was applied on the dorsal thoracic region of the larvae using a calibrated and pre-programmed automatic micro-applicator (Burkard Scientific, www.burkhardscientific.co.uk ). Twenty-five larvae were treated with each concentration (five replicates, with five larvae in each replicate). Control larvae were treated with only carrier solvent. The treated as well as the control larvae were kept on the artificial diet, and mortality was recorded after 24, 48, and 72 hr.

### Data analyses

All the data were subjected to ANOVA and transformation by arcsin data in a completely randomized design, and the means were separated using Tukey’s test. The analysis was carried out using SPSS software packages ( Gomez and Gomez 1984 ). For determination of the antifeedant index (AI50) values, the calculated data on percentage antifeedant activity were subjected to probit analysis using the Finney (1971) method and Windostat software ( windostat.software.informer.com ).

## Results

### Antifeedant activity

**Figure 1.**
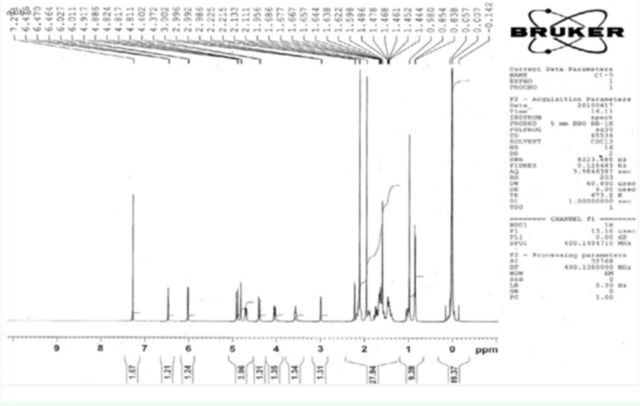
Nuclear magnetic resonance spectrometry results for clerodin. High quality figures are available online.

**Figure 2.**
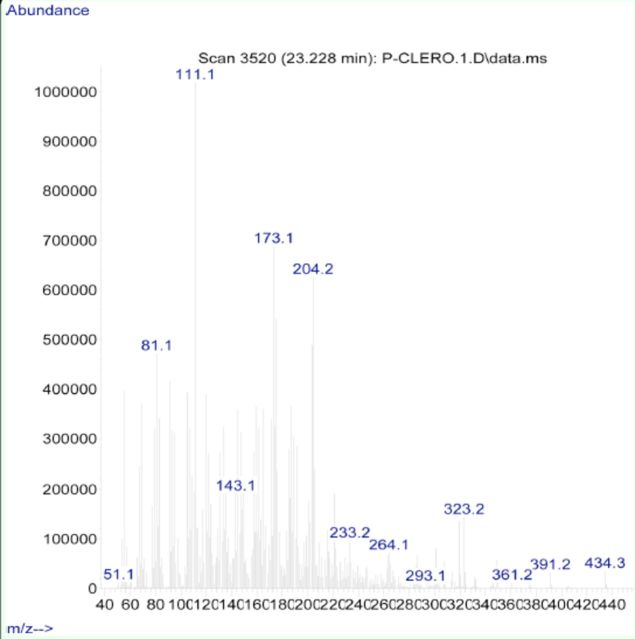
Mass spectra of clerodin. High quality figures are available online.

**Figure 3.**
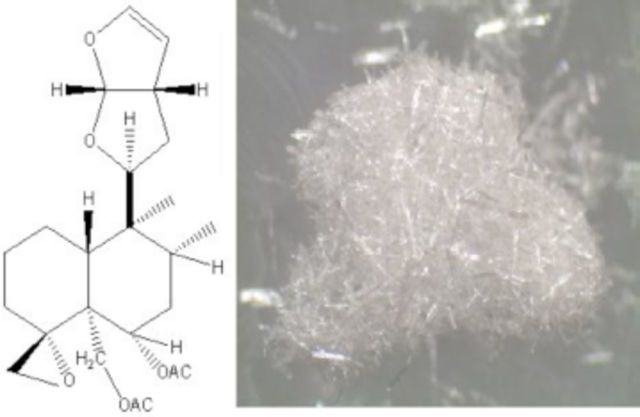
Crystals (right) and strucure (left) of clerodin. High quality figures are available online.

**Figure 4.**
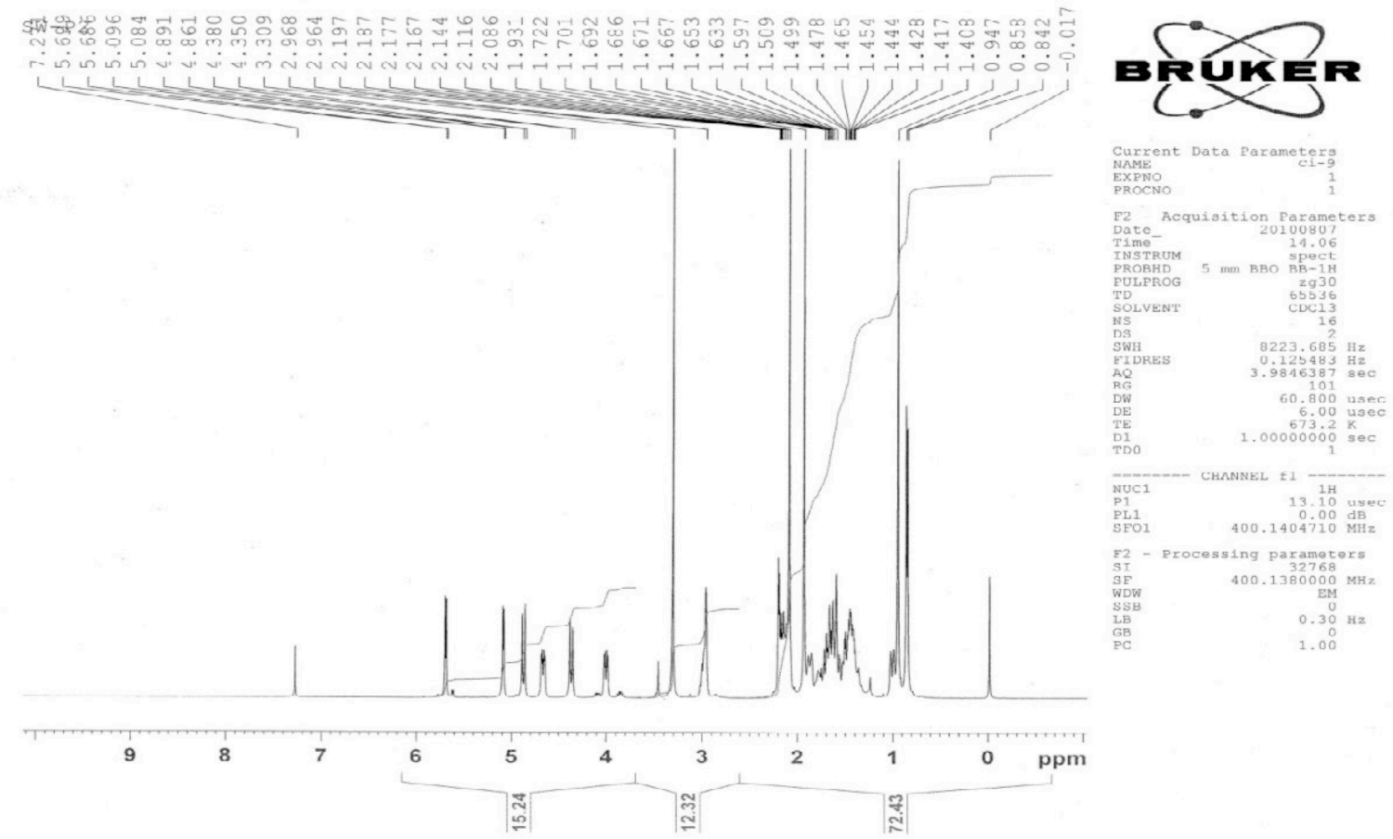
Nuclear magnetic resonance spectrometry results for 15-methoxy-14, 15-dihydroclerodin. High quality figures are available online.

**Figure 5.**
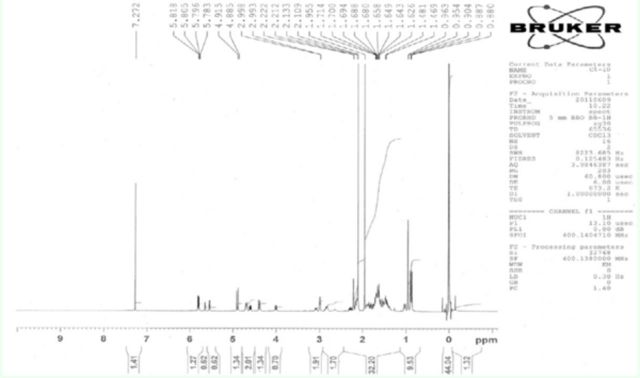
Nuclear magnetic resonance spectrometry results for 15-hydroxy-14, 15-dihydroclerodin. High quality figures are available online

**Figure 6.**
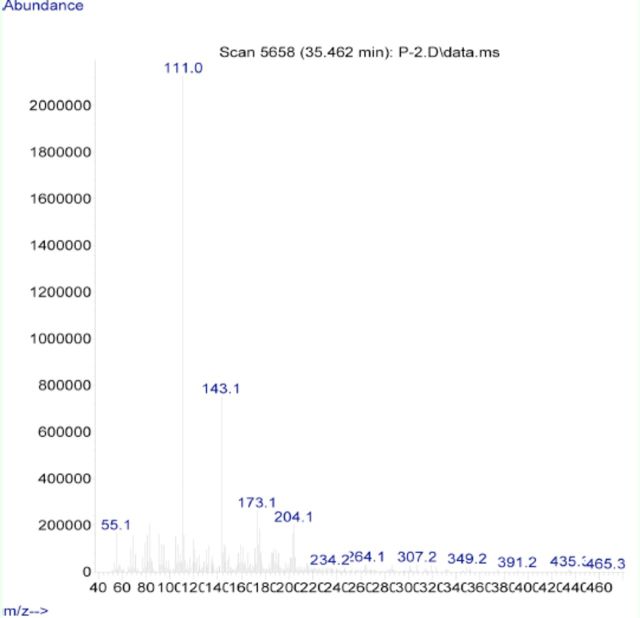
Mass spectra of 15-methoxy-14, 15-dihydroclerodin. High quality figures are available online.

**Figure 7.**
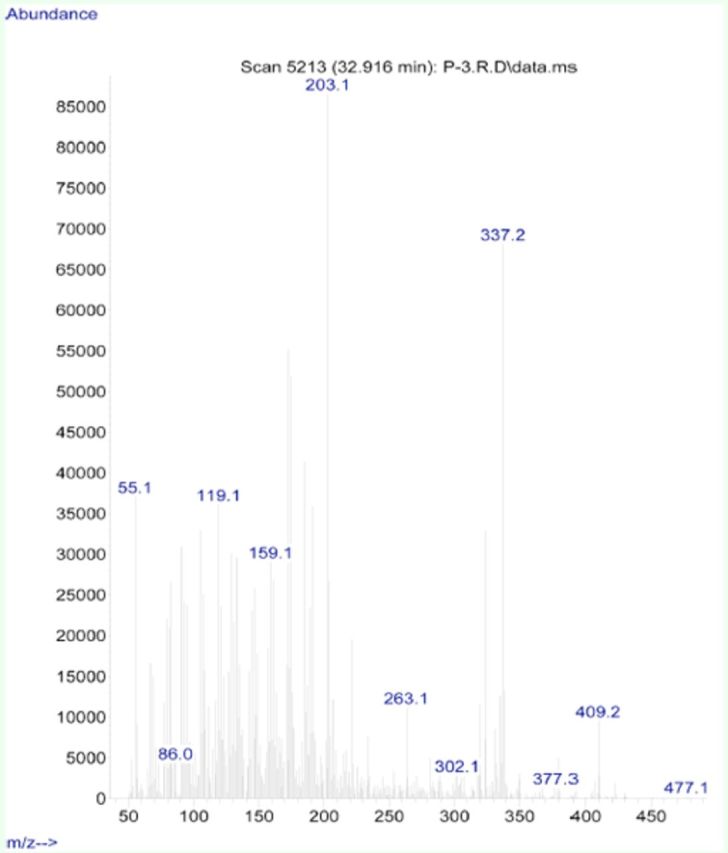
Mass spectra of 15-hydroxy-14, 15-dihydroclerodin. High quality figures are available online.

**Figure 8.**
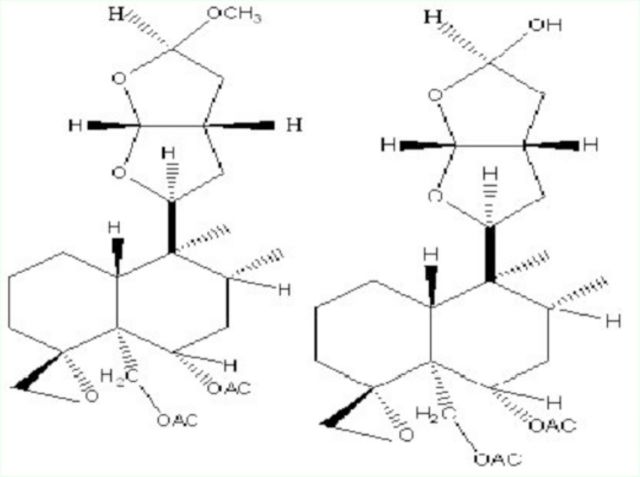
15-methoxy-14, 15-dihydrocleodin (left) and 15-hydroxy-14, 15-dihyroclerodin (right).High quality figures are available online.

The antifeedant activities of different crude extracts and fractions of *C. infortunatum* were studied using the leaf disc no-choice test method. The methanol fraction of hexane extract showed the highest antifeedant activity against *H. armigera* ( Table 1 ); activity was recorded as 95%, which was statistically significant ( *P* < 0.05) compared to other extracts, fractions, and azadirachtin. The highly-effective methanol fraction of hexane extract was dissolved in 50 mL of methanol and kept in the refrigerator for two days; crystallization occurred during this time, and crystals were then separated. The purity of the compound was examined via TLC (Rf values of 0.71, solvent system ratio of ethyl acetate to hexane was 60:40) and HPLC (RP-18 column and acetonitrile-water as the solvent system with a ratio of 70:30) at a flow rate of 0.75 mL/min that showed an Rt value of 5.683 min. Then the pure compound was subjected to NMR and MS analyses. The NMR of the compound matched with that of clerodin (CL), as it exhibited peaks characteristic of the CL molecule ( Figure 1 ). In the mass spectrum, the compound exhibited molecular ion peak [M+] at m/z 434, which was typical of the CL molecule. These spectral data confirmed the compound to be CL (Figures 2 and 3). The rest of the methanol fraction of hexane extract was subjected to column chromatography. Among the six fractions obtained, antifeedant activities of fractions 1, 2, and 3 were poor, and they were shown through TLC to be a mixture of various compounds. Fractions 4, 5, and 6 showed very good antifeedant activity with single spots in the TLC trials ( Table 2 ). Fraction 4, collected at mobile phase ratio of 75:25 (hexane:ethyl acetate), was a single compound and showed an Rf value of 0.71 (60:40 hexane: ethyl acetate) when its purity was checked by TLC, which was similar to CL. Its Rf value was confirmed by further purity analysis. Fractions 5 and 6, collected at the mobile phase ratio of 65:35 and 60:40 (hexane:ethyl acetate), respectively, were also single compounds. The purity of these compounds was checked by TLC using ethyl acetate:hexane (60:40) as the mobile phase, which showed Rf values of 0.54 and 0.31, for fractions 5 and 6, respectively. The purity was also checked by HPLC, using RP-18 column and acetonitrile-water with a ratio of 70:30 as the solvent system at a flow rate of 0.75 mL/min, which showed single peaks with Rt values of 4.933 and 1.772 min, for fractions 5 and 6, respectively. When these pure compounds were subjected to NMR and MS analyses, they exhibited molecular ion peaks at 466 and 452 (fractions 5 and 6, respectively), which corresponded to the molecular mass of the compounds ( Figures 4 and 5 ). The NMR spectra of these two compounds also matched those reported in the literature ( Figures 6 and 7 ). These spectral data confirmed that fraction 5 was 15-methoxy-14, 15-dihydrocleodin (MD) and fraction 6 was 15-hydroxy-14, 15-dihyroclerodin (HD) ( Figure 8 ).

**Table 1.**
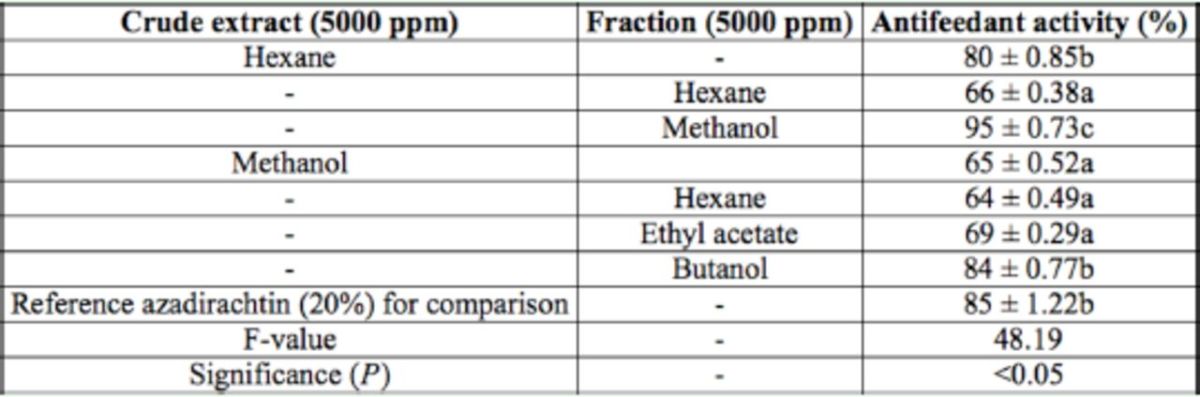
Antifeedant activity of different crude extracts and fractions of *Clerodendron infortunatum* leaves against the larvae of *Helicoverpa armigera* through no-choice test method after 24 hr. Within columns, means followed by a common letter do not differ significantly (Tukey’s test; *P* < 0.05, n = 5).

**Table 2.**
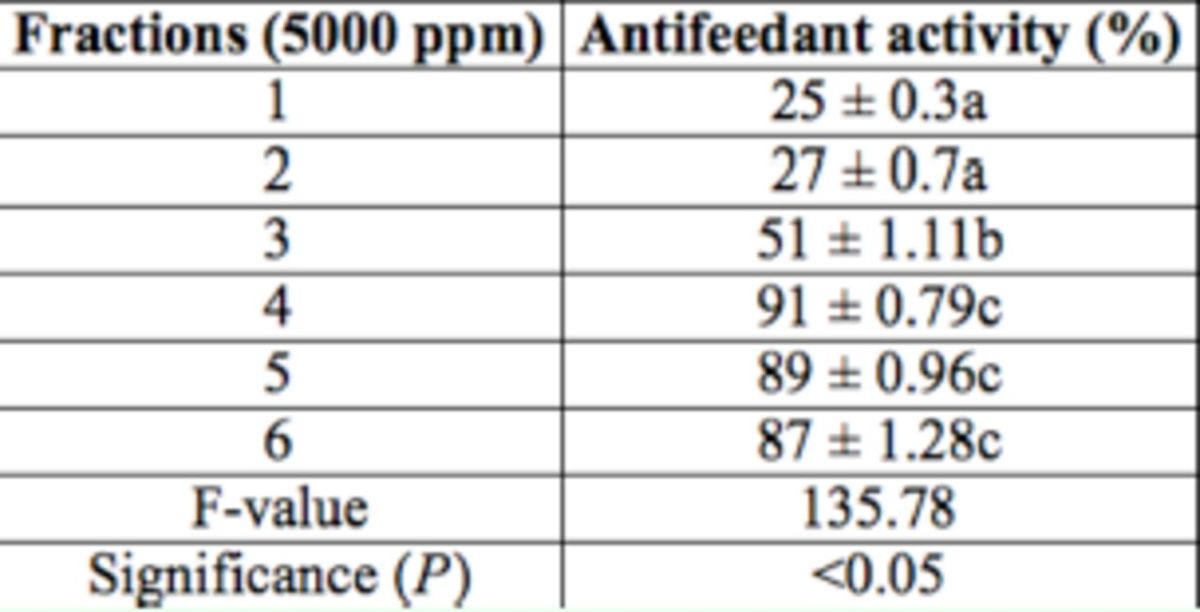
Antifeedant activity of different fractions isolated from methanol fraction of hexane extract of *Clerodondron infortunatum* leaves against the larvae of *Helicoverpa armigera* through no-choice test method after 24 hr. Within columns, means followed by a common letter do not differ significantly (Tukey’s test; *P* < 0.05, n = 5).

In the present study, 2.2 g CL, 313 mg MD, and 1.7 g HD were isolated from 2.5 kg dried leaves. The antifeedant activity of the isolated compounds are presented in Tables 3–6 . In the no-choice test condition 24 hr after treatment, at a concentration of 5,000 ppm, CL and MD resulted in 91.54 and 89.35% antifeedant activity, respectively. This activity was significantly higher than that of azadirachtin, which was 85.36% 24 hr after treatment. HD at the highest concentration showed antifeedant activity equal to that of azadirachtin. At 48 hr after treatment, CL, HD, and azadirachtin resulted in almost equal activity. AI50 values of CL, MD, and HD in the no-choice test condition 24 hr after treatment was 8, 9, and 11 ppm, respectively. In the choice test condition, all three compounds and azadirachtin showed 100% antifeedant activity at the highest concentration of 5,000 ppm after 24 hrs. AI50 values of CL, MD, and HD in the choice test condition 24 hr after treatment was 6, 6, and 8 ppm, respectively. Activity decreased when observation time was increased to 48 hr, but there was no significant difference among the activity of the compounds ( Table 7 ).

**Table 3.**
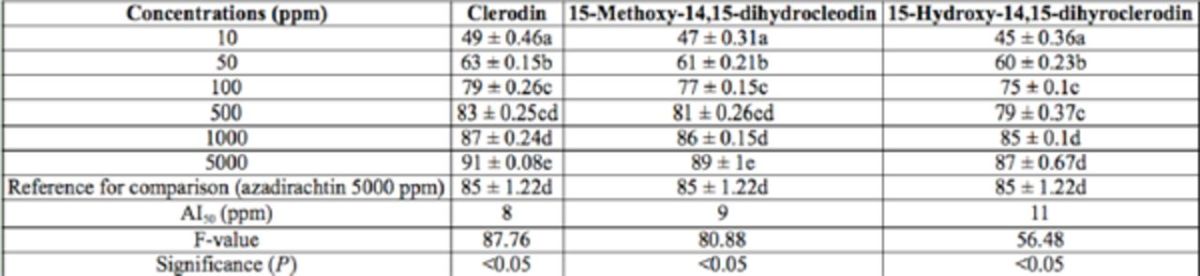
Antifeedant activity of pure compounds isolated from *Clerodendron infortunatum* against the 3rd instar larvae of *Helicoverpa armigera* through no-choice test method after 24 hr. Within columns, means followed by a common letter do not differ significantly (Tukey’s test; *P* < 0.05, n = 5).

**Table 4.**
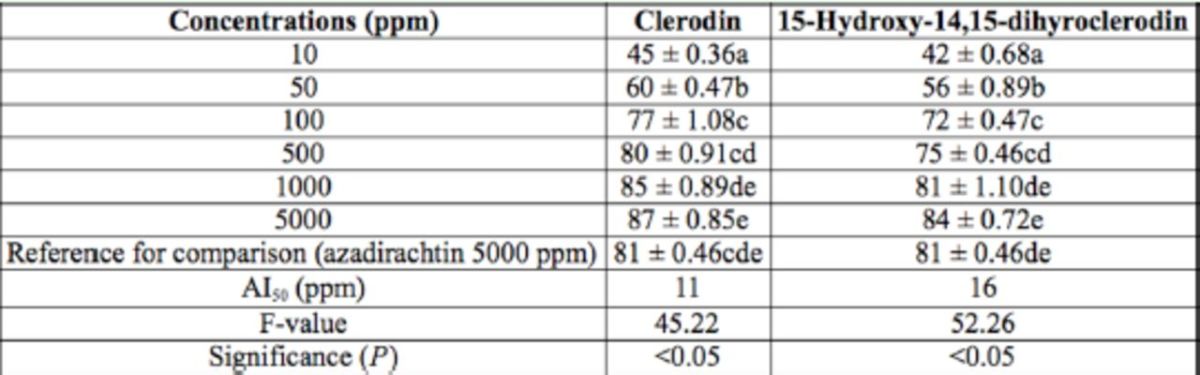
Antifeedant activity of pure compounds isolated from *Clerodondron infortunatum* against the 3rd instar larvae of *Helicoverpa armigera* through no-choice test method after 48 hr. Within columns, means followed by a common letter do not differ significantly (Tukey’s test; *P* < 0.05, n = 5).

**Table 5.**
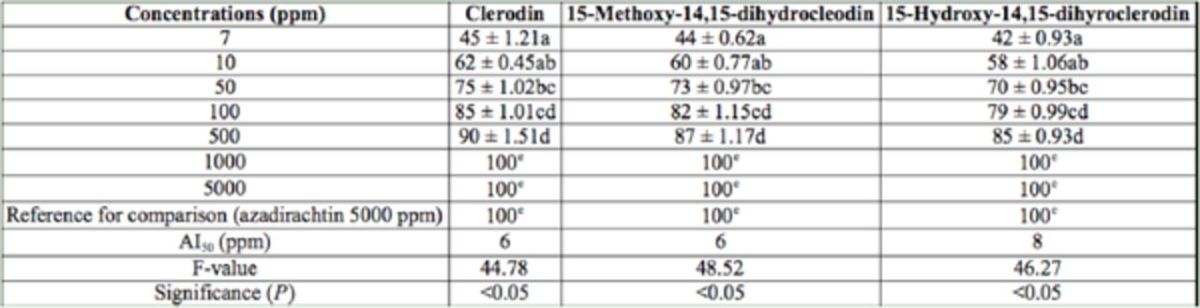
Antifeedant activity of pure compounds isolated from *Clerodondron infortunatum* against the third instar larvae of *Helicoverpa armigera* through choice test method after 24 hr. Within columns, means followed by a common letter do not differ significantly (Tukey’s test; *P* < 0.05, n = 5)

**Table 6.**
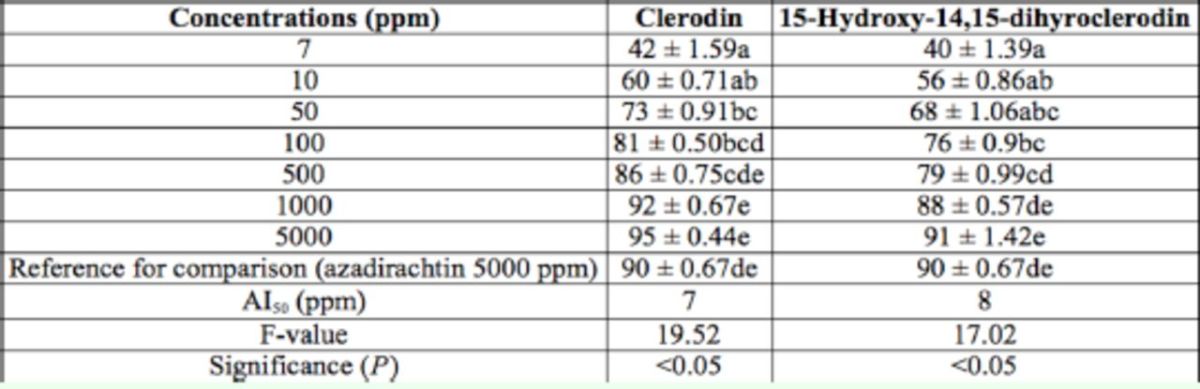
Antifeedant activity of pure compounds isolated from *Clerodondron infortunatum* against the third instar larvae of *Helicoverpa armigera* through choice test method after 48 hrs. Within columns, means followed by a common letter do not differ significantly (Tukey’s test; *P* < 0.05, n = 5).

**Table 7.**
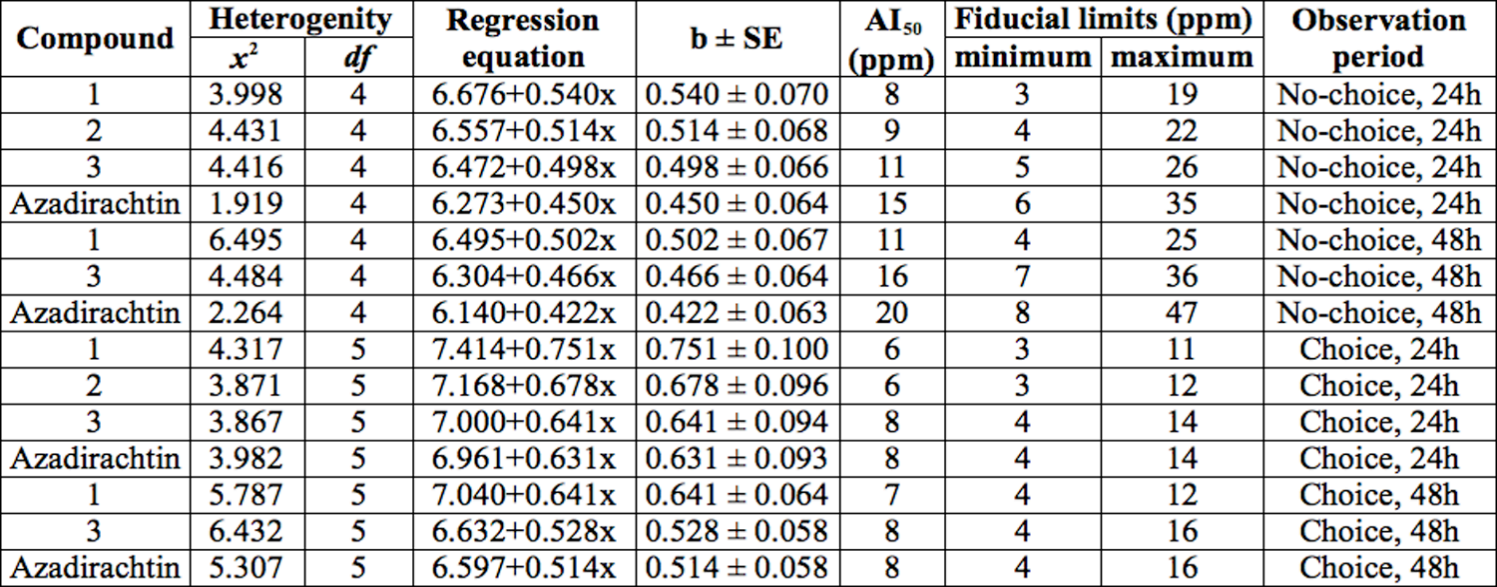
Comparative statistical results of isolated clerodane diterpenoids.

### Insecticidal activity

Very low mortality was observed when the isolated compounds were applied topically to 3 ^rd^ instar larvae of *H. armigera* ; the mortality rate was not significant compared to that of the control larvae.

## Discussion

The antifeedant activity was directly proportional to treatment concentrations in all conditions. The isolated pure compounds belonged to clerodane diterpenoids and showed very high antifeedant activity against *H. armigera* larvae. Kato et al. (1972) reported that terpenes were antifeedants extracted from *C. tricotomum* ; they isolated a number of compounds of the diterpene class with varying degrees of deterrent against *Spodoptera litura.*Koul (1982) showed that the most active compounds among diterpenes were those with a clerodane skeleton, such as CL, dihydroclerodin, and clerodin hemiacetal from *C. divaricate* , which exhibited 100% antifeedant activity at 50 ppm concentration. These results support the high antifeedant activity of pure isolated compounds that was observed in the present study. Diterpenes also provide insect resistance for plants through feeding inhibition and interference with metabolic processes essential to insect growth ( Wagner et al. 1983 ). Antifeedant activity of diterpenes was also reported by Simmonds (1996) , who used behavioral and electrophysiological bioassays to test 29 clerodane-type diterpenoids isolated from a species of *salvia* growing in Mexico, or analogues of them, for antifeedant activity against *S. littorallis* larvae. Eight of the compounds showed potent antifeedant activity in choice and no-choice bioassays, and these compounds also stimulated dose-dependent responses in the lateral styloconic of *S. littoralis* . Similarly, Cifuente et al. (2002) studied the antifeedant activity of clerodane diterpe-noides from *Baccharis sagittalis* . They isolated two clerodane-type diterpene glycoside esters and reported good antifeedant activity against *Tenebrio molitor* using a no-choice test method. The results of the present study are also supported by the studies of Kumari et al. (2003) , who isolated neocler-odane diterpenoids from *Clerodendron* spp *.* , including clerodendrin B, 3-epicaryoptin, 15-hydroxyepicaryoptin, and CL. They reported a very effective antifeedant activity against *S. litura* . In the current study, the methanol fraction of hexane extract showed higher antifeedant activity than the isolated pure compounds, which could be due to the synergistic effect of diterpenoids.

For different plant materials, various effects such as antifeedant activities, growth inhibition, and insecticidal activities have been reported (Giordano et al. 2001; Krishna et al. 2003 ; Nishida et al. 2004 ; Gonzalez-Coloma et al. 2005 ). Although the phytochemicals of *C. infortunatum* had a high antifeedant activity against *H. armigera* , when these compounds were applied topically on 3 ^rd^ instar larvae of *H. armigera* , very low mortality was observed compared to the control larvae. These results suggest that extracts of *C. infortunatum* could be used by local farmers to control *H. armigera* populations due to the powerful antifeedant activity of the compounds present in the extracts. This antifeedant activity is mainly due to the presence of clerodane diterpenoids, which could be used for development of new pesticides.

## Abbreviations

CL: clerodin
HD: 15-hydroxy-14, 15-dihyroclerodin
HPLC: high-performance liquid chromatography
MD: 15-methoxy-14,15-dihydrocleodin
MS: mass spectrometry
NMR: nuclear magnetic resonance spectrometry
TLC: thin layer chromatography
